# Navigating Grief in Wartime: Needs Assessment and Help-Seeking Behaviors among Bereaved in Ukraine

**DOI:** 10.1192/j.eurpsy.2025.1986

**Published:** 2025-08-26

**Authors:** I. Leshchuk, ?. But, Y. Zelenko, O. Khaustova, O. Chaban, I. Frankova

**Affiliations:** 1 Bogomolets National Medical University, Kyiv, Ukraine; 2 Vrije Universiteit Amsterdam; 3 ARQ National Psychotrauma Centre, Amsterdam, Netherlands

## Abstract

**Introduction:**

Traumatic losses are prevalent during armed conflicts and known to be a risk factor for prolonged grief disorder (Shevlin et al., 2024). Social support and psychoeducation are protective factors for prolonged grief disorder (Al-Gamal et al., 2018). Ukrainians are known to avoid seeking help or support after exposure to traumatic events (Quirke et al., 2021). Not much is known about help-seeking and supporting behavior following the loss of a loved one amidst the ongoing war in Ukraine.

**Objectives:**

The aim of this exploratory study was to assess the experiences, needs, and help-seeking behavior of those in bereavement and those supporting them during the war in Ukraine.

**Methods:**

An online questionnaire was designed by co-authors; data was collected in November 2023 via phone calls among users of the Opinion Internet panel by Factum Group Ukraine. Inclusion criteria were: a) Ukrainians aged 18-55; b) Internet users residing in Ukrainian cities with more than 50,000 residents before the full-scale invasion; c) those who currently live in Ukraine or abroad. The survey evaluated respondents’ experiences of enduring the loss of a loved one and supporting persons in grief after the full-scale invasion of Ukraine, as well as the need for support and information about the grieving process.

**Results:**

Fifty-three percent of survey respondents (n=400) were female. A quarter of the respondents lost a loved one since February 24, 2022 (15% due to death, 14% due to violent death, 5% missing in action), while 9% refused to respond.

Among those in bereavement, the majority (63%) reported that it was vital for them to better understand their own grief process. One-third reported the need for additional support in coping with the loss. One-fifth (21%) of those experiencing loss sought support as the experience became unbearable. Among those seeking professional care, there was a difference in the frequency of approaching different specialists. Twenty percent of the respondents reported that they did not seek help but were willing to.

The majority of respondents (62%) reported supporting a person who experienced loss. However, only 49% of them felt confident enough to do so. The majority (82%) of respondents said that they considered it important to have information on ways to support loved ones in grief.

**Conclusions:**

Despite the fact that a third of people required support and care while experiencing a loss, only 1 in 5 approached mental health professionals or other healthcare specialists. Half of those who wanted to support their loved ones did not feel confident enough. Raising awareness among the population about the process of coping with loss, normalizing seeking support, and providing possible options for supporting people in grief is of high importance for the people of Ukraine affected by the full-scale war.

**Disclosure of Interest:**

None Declared

